# Physics-Based Simulation of Soft-Body Deformation Using RGB-D Data

**DOI:** 10.3390/s22197225

**Published:** 2022-09-23

**Authors:** Daeun Kang, Jaeseok Moon, Saeyoung Yang, Taesoo Kwon, Yejin Kim

**Affiliations:** 1 Department of Computer Science, Hanyang University, Seoul 04763, Korea; 2 School of Games, Hongik University, Sejong 30016, Korea

**Keywords:** physics-based simulation, soft-body deformation, RGB-D data, topological editing, object tracking

## Abstract

Providing real-time interaction in an immersive environment has drawn considerable attention in the virtual training fields. Physics-based simulations are suitable for such environments; however, they require the definition and adjustment of coefficients that determine material properties, making the methods more complex and time-consuming. In this paper, we introduce a novel approach to simulating the soft-body deformation of an observed object. Using an off-the-shelf RGB-D sensor, the proposed approach tracks an object’s movement and simulates its deformation in an iterative manner. Polygonal models with different resolutions are used to improve the simulation speed and visual quality. During the simulation process, a low-resolution model is used for surface deformation using neighboring feature points detected from the sensor, and a volumetric model is added for internal force estimation. To visualize the observed model in detail, the deformed and low-resolution model is mapped to a high-resolution model using mean value coordinate interpolation. To handle topological deformations, such as cutting or tearing, a part intersected by a cutting tool is recognized by the sensor and responds to external forces. As shown in the experimental results, our approach generates convincing deformations of observed objects in real time.

## 1. Introduction

In recent years, virtual reality (VR) and augmented reality (AR) have been widely used in various industries. Providing interactions between an observed object and a virtual model in an immersive environment in real time has drawn considerable attention in the virtual training fields [1]. For example, the use of virtual training simulations enables users to practice operations in an efficient and safe way [2,3]. However, most current systems simulate only flat and solid objects. A training environment with more generalized objects requires visual realism and a fast response of soft-body deformation.

Previous studies simulating virtual deformations of a real object adopted a method of inverse kinematics (IK) to control high degrees of freedom in the object’s movement. A physics-based method requires the definition and adjustment of coefficients that determine material properties, making it more complex and time-consuming. Nevertheless, physics-based simulations are more suitable for use in an interactive environment in which an observed object responds to the external forces applied by a user and deforms accordingly. Recent advances in RGB-D sensors have made it easier to track an observed object in real time. However, only a few studies have simulated the soft-body deformation of a real object by using a physical approach with real-time tracking.

**Figure 1:**
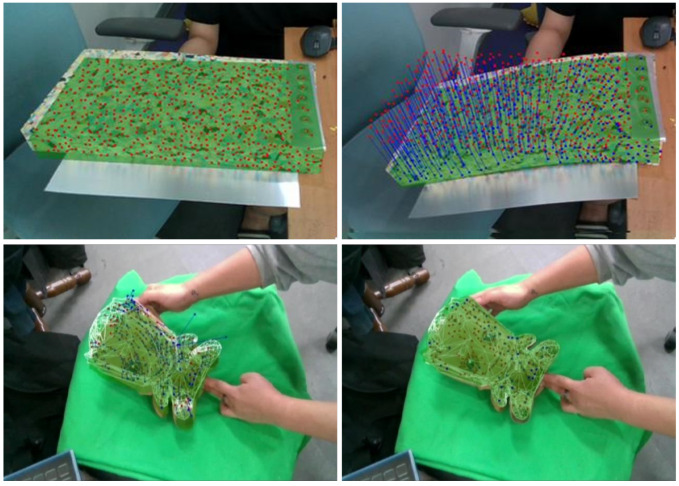
Soft-body deformation of observed objects.

In this paper, we introduce a novel approach to simulating the soft-body deformation of an observed object. Using a single RGB-D sensor, the proposed approach tracks an object’s movement and simulates its deformation simultaneously. Polygonal models with different resolutions are used to improve the simulation speed and visual quality; a low-resolution and volumetric model is used for the physics-based deformation of an observed object and a high-resolution model is used for the visual output. To handle topological deformations, such as cutting or tearing, a part intersected by a cutting tool is recognized by the sensor and responds to external forces. As shown in Figure 1, our approach generates a convincing deformation of soft-body objects in real time.

Our approach makes two major contributions to the literature. First, we introduce a physics-based approach for generating soft-body deformation using RGB-D data, which have not been actively studied in the related field. To the best of our knowledge, there has been no real-time (that is, over 30 fps) approach that performs the soft-body deformation of a real object using a single RGB-D sensor. Second, we combined several existing methods to tackle the problems of physics-based simulations. During the physical force estimation, we reformulated the problem of the internal force calculation as a linear system, which is easier to solve and implement than in previous approaches. Simulating a soft-body object with a 3D model is a complex and time-consuming process because of the force estimation required for a large number of vertices. To achieve real-time performance, we propose a simulation process that utilizes different polygonal models: a low-resolution model with a corresponding volumetric model for soft-body simulation and a high-resolution model for visual output. Overall, our approach can be utilized in various VR and AR applications that track soft-body objects in a cost-effective way.

We have organized the paper as follows: In Section 2, we review previous research on object tracking and physics-based simulation of non-rigid objects. Section 3 summarizes our approach. Section 4 and Section 5 describe the tracking and simulation processes, respectively. We present the experimental results in Section 6 and draw conclusions about our approach with its limitations in Section 7.

## 2. Related Works

### 2.1. Non-Rigid-Object Tracking

The tracking of a non-rigid object from a sequence of RGB images has been widely used in 3D model reconstruction. Provided with a template model in the rest pose, some researchers have proposed a dense approach that analyzes pixel appearance and optimizes the similarity between the target and template models [4,5,6]. Others have introduced a feature-based approach that uses shape reconstruction [7,8,9,10]. To match feature points detected between input images, a graph-based approach has been widely adopted, and it defines the geometric relationships between feature points [11,12,13,14]. By contrast, our approach introduces a physics-based simulation of soft-body deformation.

The availability of off-the-shelf RGB-D sensors [15,16] enables users to efficiently capture images of real objects using RGB and depth data. These devices have been actively researched for the detection and tracking of objects for various applications. Ren et al. used the Kinect sensor to recognize hand gestures based on novel shape distance metrics [17]. Patil and Bailke applied the RealSense sensor in order to recognize facial expressions based on a multi-layered neural network for classification [18]. For a performance animation, Lee and Kwon presented a physics-based interactive system that used a sequence of user poses as input data and simulated the user’s actions via control parameters [19]. Siena et al. reviewed the extensive use of RGB-D sensors in the medical field, particularly for measuring health outcomes [20]. While these approaches focus on tracking the motion of the human body, our approach was designed to track the deformation of soft-body objects in general.

### 2.2. Physics-Based Simulation of Non-Rigid Objects

Since the pioneering work of Terzopoulos et al., who introduced an elastically deformable model based on physical dynamics [21], there have been several studies on the simulation of different non-rigid objects, such as rubber, cloth, paper, and objects with flexible characters. The finite element method (FEM) is a popular method for estimating the physical dynamics of a soft-body object with a set of volumetric meshes by solving partial differential equations [22,23].

Some researchers have attempted to improve the robustness and quality of FEM simulations; Irving et al. demonstrated the deformation of plastic solids using numerous inverted tetrahedral meshes [24]. Subsequently, they proposed a numerical method for modeling incompressible and elastic materials [25]. Bargteil et al. improved the performance of the FEM by updating the linear basis functions and introducing a high-quality finite-element mesh for plastic deformation [26]. Kim and Pollard proposed a fast simulation system for deformable characters by adopting non-linear finite elements and a linear time algorithm for skeleton-based dynamics that can be computed in parallel on a GPU in real time [27].

To improve the performance of the FEM, a linear strain model or precomputation of a stiff matrix is used for fast and stable deformation. Müller et al. handled large deformations by estimating a tensor field that describes local rotations at every time step [28]. Nesme et al. implemented a linear FEM to deform elastic solids by using the rotational invariance and robustness of tetrahedral meshes [29]. Furthermore, linear models with a precomputed matrix or reduced dimensionality have been applied to interactive applications [30,31,32]. Unlike these approaches, the proposed approach simulated soft-body deformation from the observed data captured from an external sensor.

### 2.3. Tracking and Simulation of Non-Rigid Objects

Similarly to our approach, few studies have tracked soft-body objects and simulated their deformations in 3D space. Haouchine et al. introduced a novel framework for building a 3D virtual model and simulating the observed deformation using stereographic images [33]. However, this was not in real time, and they used synthetic dataset, while our approach observes and simulates real objects in real time. Leizea et al. tracked object deformation using a single RGB-D sensor in real time [34]. In their approach, a mass–spring system was applied to deform the observed objects, and this was less accurate than an FEM-based approach such as ours. Using an FEM-based framework, Petit et al. tracked multiple soft-body objects without textures [35]. By contrast, our approach tracks a set of features on textured objects, and this is more general for real objects. Sengupta et al. proposed a physics-based approach for simulating deformable objects using an RGB-D sensor [36]. However, it operated at an interactive speed (that is, under 30 fps) and did not support topological deformation, which is useful in various applications [2].

## 3. Overview

**Figure 2:**
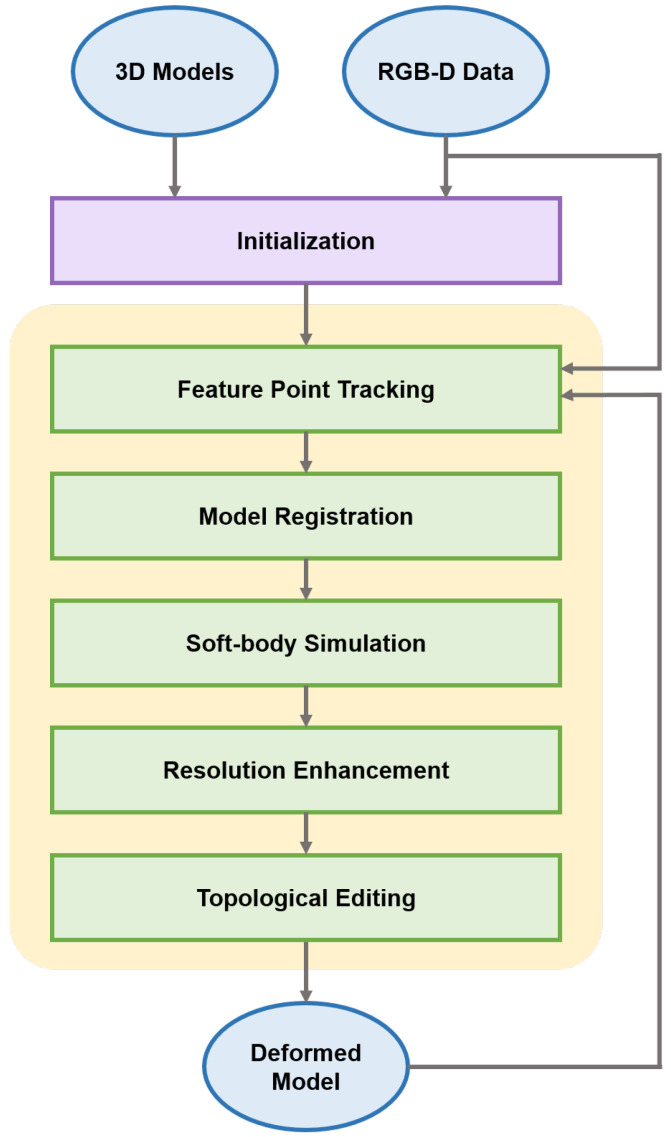
Overview of the proposed approach.

Figure 2 shows a brief overview of the proposed approach. The proposed simulation process is summarized as follows:

- Initialization: A set of polygonal 3D models are prepared for the deformation of an observed object. These include a low-resolution model with a volumetric model for soft-body simulation and a high-resolution model for the visual output. A sequence of RGB-D images is continuously provided by a sensor.
- Feature-point tracking: In the initial frame, a set of feature points are detected from RGB images using a local feature detector. For every frame, these points are tracked as feature vectors between two adjacent frames.
- Model registration: To set the correspondences between the feature points and surface vertices in the model, a low-resolution model is coordinately aligned using an iterative closest point method. For a smooth deformation, each feature point controls the surface vertices using weight values that are assigned to each vertex.
- Soft-body simulation: To determine the deformation of a soft-body model, the internal force is estimated by applying a physical force to the volumetric model. A projective dynamics method is adopted to accelerate the force calculation for each vertex.
- Resolution enhancement: To visualize the observed object in high detail, the deformed model in low resolution is mapped into a high-resolution model. During the mapping process, a weight-based interpolation method is used to control the surface vertices in the high-resolution model.
- Topological editing: To handle topological deformations, such as cutting or tearing, a cutting tool’s trajectory is tracked by a sensor. The intersected part is reshaped to a triangulated model using Delaunay triangulation, representing a new surface of the model.

## 4. Object Tracking

Our approach simulates object deformation by tracking a set of feature points from a sequence of RGB images and mapping their movements to a low-resolution (3D polygonal) model. In the initial frame, the model is registered to a space of feature points, and the feature points are mapped to the surface of the model. For every updated frame, a fast feature detector is used to track the positional changes of the feature points.

### 4.1. Initialization

**Figure 3:**
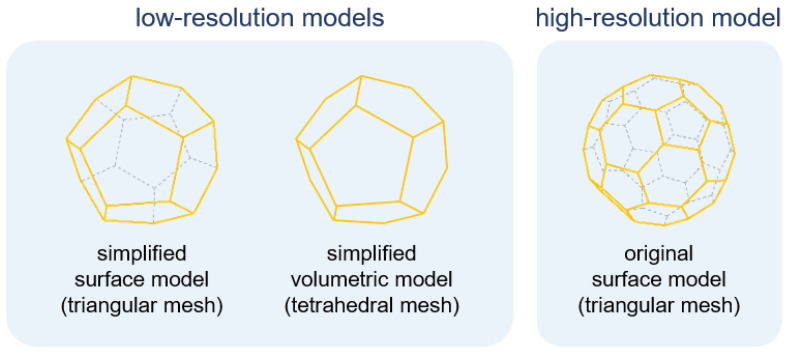
Different object models used for simulation (low-resolution models) and visualization (high-resolution model).

The default shape of the model is created from an object in the rest state without any applied force. For each object, three types of polygonal models are prepared for different purposes: a low-resolution model and a volumetric model for the simulation and a high-resolution model for visualization. As shown in Figure 3, the low-resolution model is a simplified version of the high-resolution model, and both models are composed of a set of triangular polygons. The volumetric model consists of a set of tetrahedral polyhedrons, which are placed inside the low-resolution model and used for internal force estimation. The low-resolution model is mapped to a high-resolution model to represent the deformation in high detail.

### 4.2. Feature-Point Tracking

A set of feature points are detected and tracked from RGB images that are streamed from a sensor using a local feature detector. The oriented FAST and rotated BRIEF (ORB) method is used to recognize corner points from the given images by finding pixels that are darker than their surrounding pixels. The positional changes of these points are tracked between two consecutive frames by comparing the main pixels with the surroundings [37]. We refer to the differences between two matching feature points in different frames as *feature vectors*. Compared to other feature detection methods, ORB shows better performance and accuracy in the point-matching process and is suitable for use in real time.

**Figure 4:**
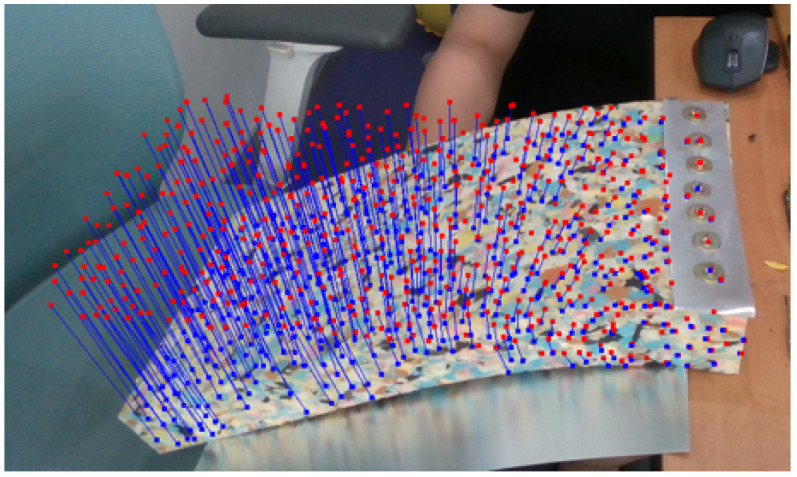
Feature points detected at the initial (red points) and current (blue points) frames are tracked as feature vectors (blue lines) during the tracking process.

For each frame, the positional changes of feature points are tracked as 3D points. A sequence of RGB images with the corresponding depth data (in the form of depth images) is provided by the RGB-D sensor. Thus, the coordinates of the feature points in the low-resolution model can be easily assigned from the depth data. Figure 4 shows feature points that are tracked as feature vectors during the tracking process.

### 4.3. Model Registration

#### 4.3.1. Coordinate Alignment

The positional changes of feature points control the degree of deformation. As deformation is represented by the surface of the low-resolution model, it is necessary to set the correspondences between the feature points and vertices. Such a coordinate alignment problem can be solved by finding a rigid transformation **T**, which transforms one coordinate system into another. If the coordinates of feature points are selected as a reference system, **T** can be estimated using the iterative closest point (ICP) method [38,39], which minimizes the error function, $E\left(T\right)=E\left(T_{R},T_{L}\right)$, where $T_{R}$ and $T_{L}$ are the rotation and translation of the vertex data with respect to the reference system, respectively.

Let $p_{i}$ be the *i*th feature point, where $i\in [1,\cdots ,N_{p}]$, and let $N_{p}$ be the total number of feature points detected from the image sequence. E(·) is evaluated as follows:

(1) $$E\left(T_{R},T_{L}\right)\propto \frac{1}{N_{p}}\sum_{i=1}^{N_{p}}\left|\right|p_{i}-R_{N}\left(T_{R}v_{i}+T_{L}\right){\left|\right|}^{2},$$

where $v_{i}$ denotes the corresponding vertex in the low-resolution model. $T_{L}=\bar{p}-T_{R}\bar{v}$, where $\bar{p}=\frac{1}{N_{p}}\sum_{i=1}^{N_{p}}p_{i}$ and $\bar{v}=\frac{1}{N_{p}}\sum_{i=1}^{N_{p}}v_{i}$. The correlation matrix **C** between $p_{i}$ and $v_{i}$ is evaluated as

(2) $$C=\sum_{i=1}^{N_{p}}\left(p_{i}-{\bar{p}}_{i}\right){\left(v_{i}-{\bar{v}}_{i}\right)}^{⊤}={UCV}^{⊤}.$$

The optimal solution for E(·) is $T_{R}={UV}^{⊤}$ with $C={UCV}^{⊤}$ derived from single-value decomposition (SVD). Furthermore, $R_{N}$ is a rotation between $p_{i}$ and $v_{i}$, and it is manually set and used to improve the convergence speed of the ICP. We empirically set $N_{p}$ to 1000, which is adjustable. This coordinate alignment is performed only once in the initial frame.

#### 4.3.2. Correspondence Mapping

The object model is deformed by controlling the vertices on the surface of the low-resolution model. For each vertex, a set of neighboring feature points is assigned, and distance-based weights are estimated for smooth vertex control. A K-nearest neighbor approach with inverse distance weighting (KNN-IDW) is applied to every vertex. For $v_{i}$, the maximum of the $N_{k}$ nearest feature points is selected within the distance threshold $\delta_{k}$. The weight of the *j*th nearest feature point for $v_{i}$ is estimated as follows:

(3) $$w_{i,j}=\frac{1}{D(v_{i},p_{j})}-\frac{1}{D(v_{i},p_{k})},$$

where $1\leq k\leq N_{k}$, $p_{j}$ denotes the *j*th feature point in $\left\{p_{1},p_{2},\cdots ,p_{k}\right\}$, which are sorted in the order of $v_{i}$, and D(·) measures the Euclidean distance between two points. It is noted that $w_{i,j}$ is normalized such that $\sum_{j=1}^{k}w_{i,j}=1$. This weight estimation is performed only once in the initial frame.

**Figure 5:**
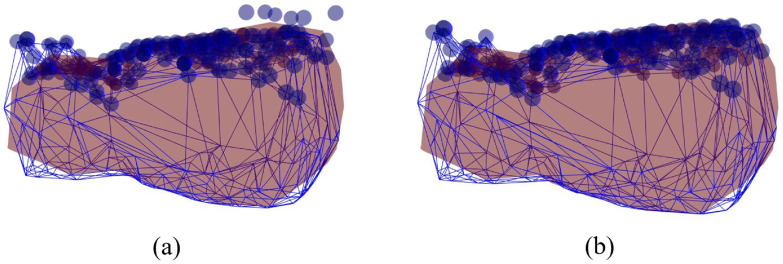
Feature points (blue points) on the low-resolution models (blue lines) that are mapped from (**a**) an initial frame to (**b**) an updated frame.

For updated frames, the position of $v_{i}$ is estimated as follows:

(4) $$v_{i,t}=v_{i,t-1}+\sum_{j=1}^{k}w_{i,j}p_{j,t},$$

where $p_{j,t}$ is the *j*th feature point in the current frame *t*. In our approach, we set $\delta_{k}=0.01$ and $N_{k}\leq 10$, which are empirical and adjustable by a user. Figure 5 shows the positional changes of the vertices in an updated frame, where the feature points are mapped to the surface of the low-resolution model.

**Figure 6:**
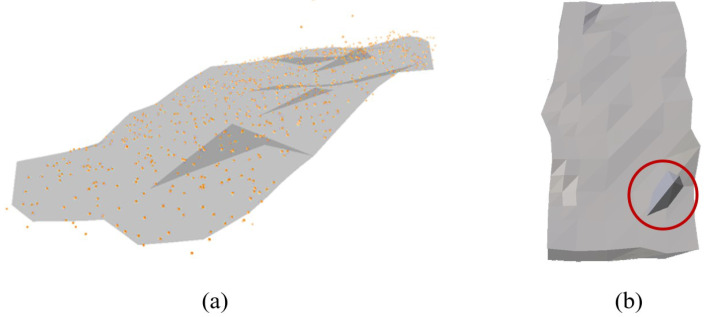
Surface deformation of a low-resolution model (**a**) using updated feature points and (**b**) showing distortion (red circle).

As shown in Figure 6, the surface of the deformed model (that is, a long cuboid-shaped sponge) appears rough and distorted owing to the feature points that are irregularly distributed. For example, the smoothness of the surface is influenced by the density of the neighboring feature points around each vertex. Surface artifacts, such as twisted or missing faces, can be observed if the feature points are occluded or skipped during the feature-tracking process. Therefore, a volumetric model is added to the low-resolution model, which maintains its original shape from an external force, as described in the subsequent section.

## 5. Soft-Body Simulation

For an accurate deformation, we apply a volumetric model to the low-resolution model. As shown in Figure 3, the volumetric model consists of a set of tetrahedral polyhedrons and introduces an internal force to preserve its original shape. To estimate the physical dynamics of a soft-body model filled with numerous tetrahedral polyhedrons, we adopt the FEM by solving partial differential equations.

### 5.1. Internal Force Estimation

For each vertex of the tetrahedral polyhedrons, the FEM determines the stresses and strains based on the changes in the positions and velocities of the vertices. Let $x_{l,t}$ and $v_{l,t}$ be the position of the *l*th vertex and its velocity at frame *t*, respectively, where $l\in [1,\cdots ,N_{v}]$, $N_{v}$ denotes the total number of vertices in the volumetric model, and $x_{l,t},v_{l,t}\in R^{3N_{v}}$. The updated position and velocity, $x_{l,t+1}$ and $v_{l,t+1}$, respectively, of the *l*th vertex at the next frame are defined as follows:

(5) $$x_{l,t+1}=x_{l,t}+hv_{l,t+1},$$

(6) $$v_{l,t+1}=v_{l,t}+ha_{l,t+1},$$

where *h* denotes the length of the time step and $a_{l,t+1}$ denotes the acceleration of the vertex at frame t+1. Using Newton’s laws of motion, Equation (6) can be rearranged as follows:

(7) $$v_{l,t+1}=v_{l,t}+hM^{-1}\left(F_{\mathrm{in}}\left(x_{l,t+1}\right)+F_{\mathrm{ex}}\right),$$

where **M** represents a sparse matrix for the mass of each tetrahedral polyhedron, $F_{\mathrm{in}}\left(\cdot \right)$ represents an internal force with the given position, and $F_{\mathrm{ex}}$ denotes an external force. This equation can be reformulated as an energy minimization problem; however, it is expensive to solve a different linear system for each iteration [40]. In our approach, the mass of the entire object model is set to 1, and the mass of each tetrahedral polyhedron is set as the proportion of the volume of each tetrahedral polyhedron to the volume of the entire object model.

Given the updated feature points in every frame, $v_{l,t}$ can be substituted with a constant value. Using the backward Euler method, $F_{\mathrm{in}}\left(\cdot \right)$ on each vertex is defined as follows:

(8) $$F_{\mathrm{in}}\left(x_{l,t+1}\right)≃F_{\mathrm{in}}\left(x_{l,t}\right)+\frac{\partial F_{\mathrm{in}}}{\partial x}{|}_{x_{l,t}}\left(x_{l,t+1}-x_{l,t}\right),$$

which is nonlinear and difficult to estimate. Therefore, using the Taylor expansion, it is reformulated as follows:

(9) $$\left[M-h^{2}\frac{\partial F_{\mathrm{in}}}{\partial x}\right]v_{l,t+1}=Mv_{l,t}+h\left(F_{\mathrm{in}}\left(x_{l,t}\right)+F_{\mathrm{ex}}\right),$$

which transforms our soft-body simulation into a problem of internal force estimation.

### 5.2. Projective Dynamics for Physical Force

The Newton’s-method-based equations are time-consuming for estimating the internal force for each vertex on the volumetric model because of nonlinearity. In our approach, a projective dynamics (PD) method is adopted to update the positional changes of a vertex in real time [41]. It is an implicit time integration method for physics-based simulations and shows a high performance in computation time by replacing nonlinear terms with linear ones.

For the position of the *i*th vertex $x_{i}$, the internal force $F_{\mathrm{in}}$ is replaced with the potential energy *S* as follows:

(10) $$F_{\mathrm{in}}\left(x_{i}\right)=-\nabla S\left(x_{i}\right),$$

where S(x) denotes the sum of the scalar functions for the elastic strain energy of the material. It is defined by the difference between *x* and its projection on the constraint manifold *y* as follows:

(11) $$S\left(x_{i}\right)=\frac{k}{2}{∥A_{i}x_{i}-y_{i}∥}_{F}^{2},$$

where ${∥\cdot ∥}_{F}$ denotes the Frobenius norm, and **A** and *k* denote a constant matrix and the non-negative weight values used to define the constraints, respectively. For more details, we refer the reader to a previous study that applied FEM simulations with PD [42].

**Figure 7:**
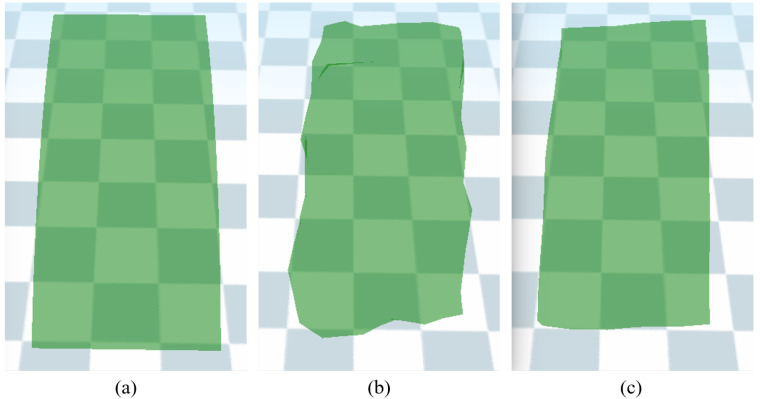
Model deformation: (**a**) initial shape, (**b**) deformed shape from updated feature vectors, and (**c**) deformed shape from the FEM simulation.

The PD method solves the optimization problem to minimize the internal force while satisfying the following constraints:

minxi+1∑i12h2∥M12(xi+1−xi−hvi−h2M−1Fex)∥F2︸T1+∑ik2∥Aixi−yi∥F2︸T2,

where the former term of the optimizing problem $T_{1}$ is the position constraint that moves a vertex to a target position, and the latter term $T_{2}$ is the co-rotational elastic constraint that preserves the mesh volume. Our approach first minimizes $T_{2}$ while keeping $T_{1}$ fixed (a local strategy in PD), and then determines the minimized *x* in $T_{1}$ by solving the pure quadratic problem with a minimum error (global strategy in PD). As shown in Figure 7, the object model with physical force exhibits more natural deformation than that updated from the feature vectors.

### 5.3. Resolution Enhancement

**Figure 8:**
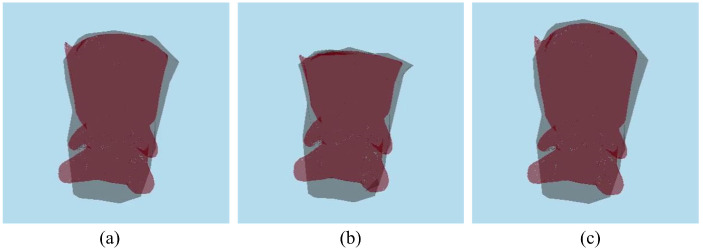
Deformation of a high-resolution model (red) enclosed by a low-resolution model (gray): (**a**) initial, (**b**) pushed, and (**c**) pulled pose.

The deformation of the observed object uses low-resolution models during the physical force estimation. Visualizing a deformed object in high detail is necessary for accurate interpretation. In our approach, the deformed model is converted into a high-resolution model by setting the mesh correspondence between the two resolution models. We adopt mean-value coordinate (MVC) interpolation, which sets a dense correspondence between the triangular faces of a low-resolution model and the vertices of a high-resolution model [43]. Unlike the KNN-IDW method used in Section 4.2, the MVC interpolation scheme additionally uses the triangular mesh topologies of both models. Since a high-resolution model is enclosed by a low-resolution model, as shown in Figure 8, the weight values for the interpolation are estimated as follows:

(12) $$w_{ij}=2\frac{tan\left[\frac{\phi_{i-1}}{2}\right]+tan\left[\frac{\phi_{i}}{2}\right]}{|x_{j}^{H}-x_{i}^{L}|},$$

where $x_{i}^{L}=\left\{x_{1}^{L},x_{2}^{L},\ldots ,x_{m}^{L}\right\}$ are vertices on the low-resolution model, $x_{j}^{H}=\left\{x_{1}^{H},x_{2}^{H},\ldots ,x_{n}^{H}\right\}$ are vertices on the high-resolution model, $w_{ij}$ denotes the weight value of $x_{i}^{L}$ for $x_{j}^{H}$, and $\phi_{i}$ denotes the angle formed by $x_{i}^{L}$, $x_{j}^{H}$, and $x_{i+1}^{L}$. It is noteworthy that Equation (12) can be estimated in parallel because of the linearity of the MVC. The updated vertices on the high-resolution model are determined as follows:

(13) $${\hat{x}}_{j}^{H}=\sum_{k=1}^{m}\lambda_{k}x_{k}^{L},$$

where $\lambda_{ij}$ is obtained by normalizing $w_{ij}$,

(14) $$\lambda_{ij}=\frac{w_{ij}}{\sum_{k=1}^{m}w_{kj}}.$$

### 5.4. Topological Editing

The proposed approach operates well for geological deformations, such as bending and pushing, but not for topological deformations, such as cutting or tearing. For topological deformations, the intersected part of the surface of the model should be recognized and filled with triangular polygons. In our approach, we assume that topological deformations are performed with a cutting tool, where its location related to the observed object is recognizable by a sensor. Given the features (i.e., a synthetic square marker) of the cutting tool in advance, we tracked its trajectory and determined where it cut the object model using a fiducial marker system [44]. For the fast-editing process, a ray–triangle interaction is used to detect the line segment intersecting the triangular polygons of the object model [45].

When the object model is cut, the triangular polygons on the intersected surface are filled with a set of triangular polygons. We reshape the intersected part using Delaunay triangulation [46]. Given multiple vertices on a 2D plane (that is, each surface polygon and cutting plane), it divides the plane into multiple triangles so that the minimum angles of each triangle have a maximum value, maintaining the lowest number of sharp-shaped triangles. In this manner, we generate triangular polygons based on vertices of the intersection without creating new vertices. Therefore, the topological deformation maintains the minimum total mesh size and operates in real time.

## 6. Experimental Results

Experiments were performed on an Intel Core i7-6700K 3.6Ghz CPU with an NVIDIA GeForce GTX 1080 Ti GPU and 16 GB of DDR4 memory on the Windows 10 operating system. An Intel RealSense D435 was used to track the observed objects, such as a cuboid sponge and a plush doll. An open-source 3D engine (i.e., OGRE) was used to render the object models. Table 1 presents the polygonal models used in the experiments. Our system is best understood through examples of its use, as described in the subsequent sections and the accompanying video located at https://drive.google.com/file/d/15wONJAiMGHz3WTCnQNn8gn8BkxHMHxG-/view?usp=sharing (accessed on 11 August 2022) .

**Figure 9:**
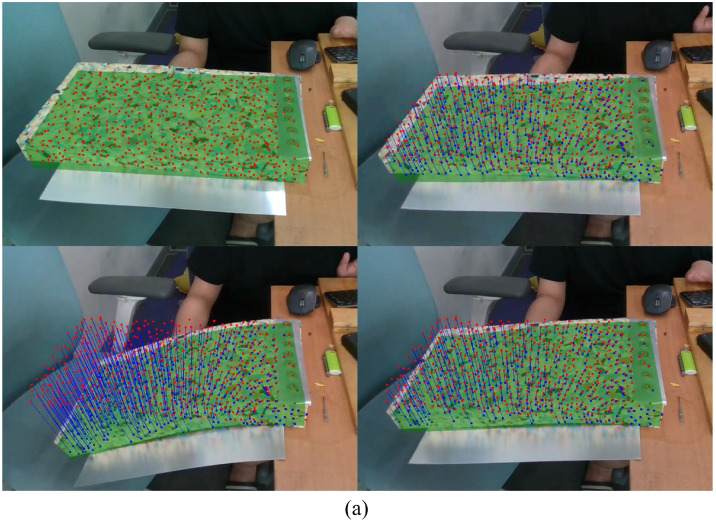

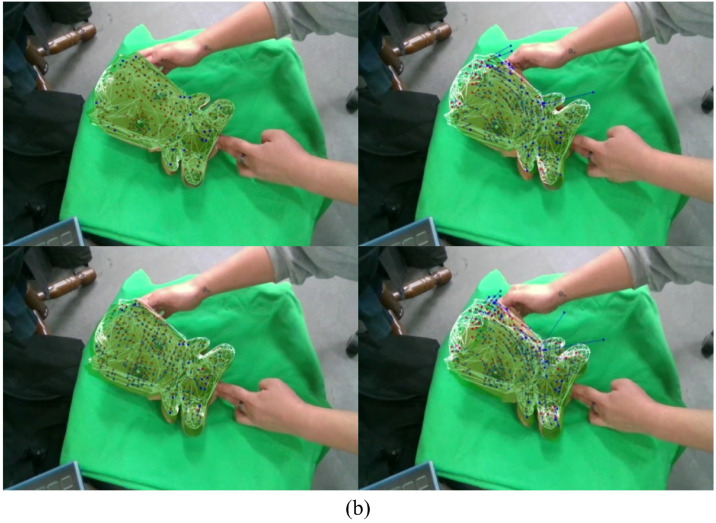
Deformation of (**a**) a cuboid sponge and (**b**) plush doll: The high-resolution model (green) was deformed by tracking the feature vectors (blue) of the feature points (red) for every frame.

As shown in Figure 9, the deformation of the soft-body models was simulated by tracking 1000 feature points detected on the observed objects. Geological deformations, such as bending and pushing, were tested, and simulation results were generated, as shown in the figures and video. Accurate results were obtained using the high-resolution model, such that the average difference between the corresponding vertex and the ground-truth model was approximately 2.56 mm. Table 2 shows that our approach simulated the deformation of object models over 30 fps and that it is capable of real-time performance. It is noteworthy that the initialization was performed only once in the initial frame, and the computation time for topological deformation was negligible, which is excluded in Table 2.

**Figure 10:**
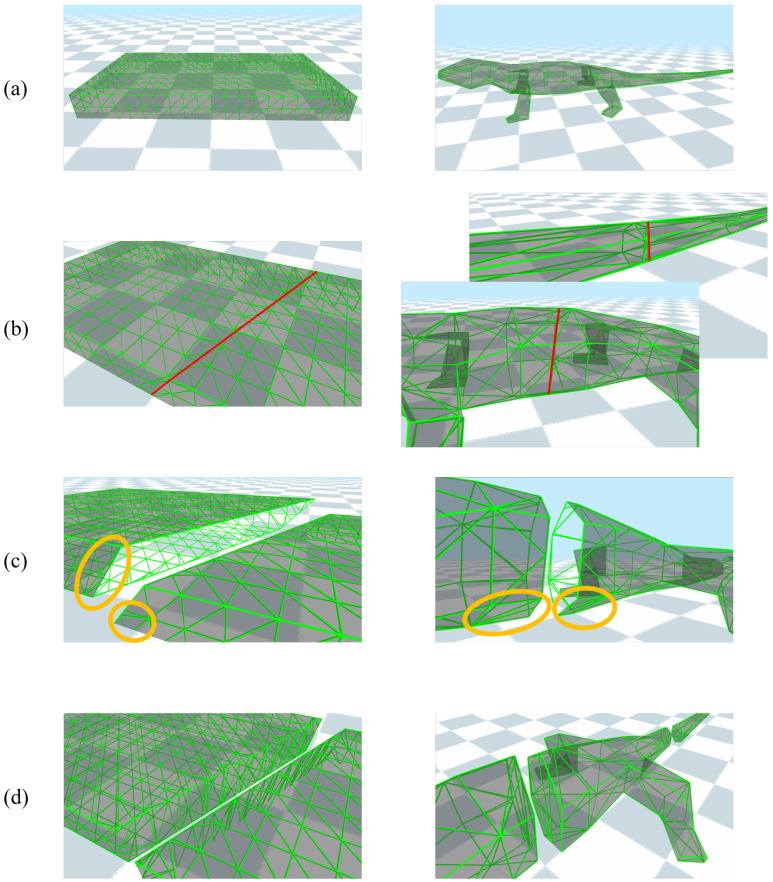
Topological deformation on a cuboid sponge model (left column) and an iguana model (right column): (**a**) original models, (**b**) mesh intersections (red lines), (**c**) deformed models without filling of the surface mesh (yellow circles), and (**d**) deformed models after filling the surface mesh.

Figure 10 shows examples of topological deformations by cutting a cuboid sponge model and an iguana-shaped model. This showed that the polygons around the intersected part included triangles with sharp angles and undesired holes on the surface. However, after reshaping the surface mesh, the triangular polygons around the intersected part became more regular. Furthermore, the intersected part was filled with a new surface mesh, which generated accurate results for cutting or tearing deformations.

**Figure 11:**
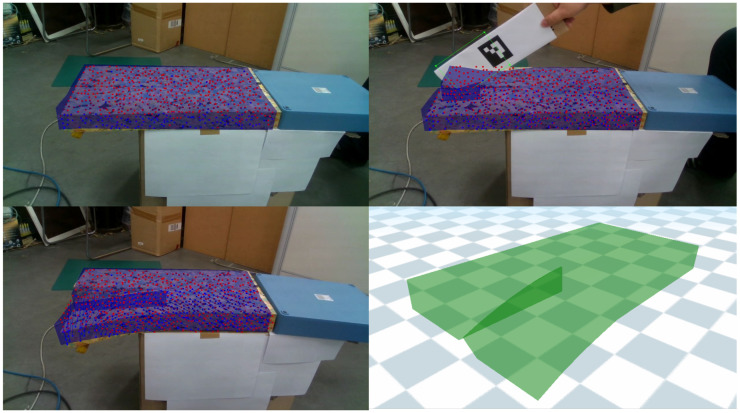
Topological deformation of a cuboid sponge: The sponge was cut by the marked board, which generated a reshaped mesh in real time.

Figure 11 shows the results of simulating the deformation of a cuboid sponge cut by a marked board in real time. While the sponge model was being cut, the feature points and board were simultaneously tracked by a sensor. To reliably track the trajectory of the cutting tool, we used the ArUco marker, which is composed of a black border and binary codification that determines its identifier [44]. While the cutting tool was traced as a rectangle, the intersection was detected between the rectangle and deformable object model. On the intersected part of the board, a new surface mesh was generated for accurate outputs. We could track the soft-body deformation and cutting tool simultaneously, which may be useful in interactive simulations, such as in medical practice or training operations.

## 7. Conclusions

In this study, we introduced a physics-based simulation of soft-body deformation using RGB-D data. Using a low-cost RGB-D sensor, our approach simulated the deformation of observed objects by tracking object movement and estimating the physical force in an iterative manner. During the simulation process, a set of models with different solutions was used to improve the simulation speed and visual quality. Unlike previous approaches, our approach handled tracking and simulation problems simultaneously by integrating several existing methods into a single framework. Furthermore, it generated realistic results for topological deformations by reshaping the intersected part. We believe that our approach is applicable to various interactive applications in the VR and AR environments.

Our approach has several limitations. The current tracking method (that is, ORB) can be affected by noisy input data. However, we expect that the physical simulation part compensates for some of inaccuracy caused by noisy data. Topological deformations require a cutting tool with a marker for robust tracking. In addition, object models with topological changes (that is, torn or separated) require a more sophisticated method to track feature points that are not seen in the initial frame. For objects with sparse feature points, the current distance error between the object model and observed object may not be sufficiently accurate for applications (e.g., medical or biological operations) that require higher precision; thus, we require a further study in order to improve the simulation accuracy.

## Figures and Tables

**Table 1:** Specifications of object models.

Model	Sponge		Doll	
Number	Vertices	Polygons	Vertices	Polygons
High-resolution	522	1040	18,650	7190
Low-resolution	132	260	128	252
Volumetric	132	300	154	494

**Table 2:** Computation time (Init.: initialization, Sim.: simulation).

Model	Sponge		Doll	
Time (ms)	Init.	Sim.	Init.	Sim.
Feature-Point Tracking	15.51	0.56	22.53	0.67
Soft-body Simulation	21.22	10.62	23.61	29.02
Resolution Enhancement	18.41	0.38	542.31	2.81
Total	55.14	11.56	588.45	32.50

## Data Availability

Not applicable.

## Abbreviations

The following abbreviations are used in this manuscript:

VR	Virtual reality
AR	Augmented reality
IK	Inverse kinematics
FEM	Finite-element method
FAST	Features from accelerated segment test
BRIEF	Binary robust independent elementary features
ORB	Oriented FAST and rotated BRIEF
ICP	Iterative closest point
SVD	Single-value decomposition
KNN-IDW	K-nearest neighbor approach with an inverse distance weighting
PD	Projective dynamics
MVC	Mean-value coordinate
